# Diagnostics of Gastrointestinal Motility and Function: Update for Clinicians

**DOI:** 10.3390/diagnostics12112698

**Published:** 2022-11-04

**Authors:** Amir Mari

**Affiliations:** Gastroenterology Department, Nazareth Hospital, Azrieli Faculty of Medicine, Bar Ilan University, Safed 16100N, Israel; amir.mari@hotmail.com or amir_mari@nazhosp.com; Tel.: +972-4-6028814; Fax: +972-602-8844

Disorders of gastrointestinal (GI) tract motility and function are prevalent in the general population and negatively affect quality of life [1]. The principle aim of this editorial is to serve as an introduction for this Special Issue, which presents the latest advances in the diagnostics of GI motility and function.

Many chronic symptoms may originate from functional GI disorders, including those affecting the upper esophageal sphincter (UES), esophageal peristalsis, lower esophageal sphincter (LES), gastric emptying, small intestinal motility and absorptive function, colonic transit, and colonic dysbiosis, as well as disorders related to the anorectal region such as dyssynergia and defects of the anal sphincter [2,3]. Clinically, these disorders may present with a wide range of symptoms including globus sensation, dysphagia, gastroesophageal reflux symptoms, dyspepsia, abdominal pain, bloating, and defecatory disorders such as diarrhea or constipation [3]. These symptoms contribute to a significant proportion of patients seeking medical care from general physicians and for referrals to specialist gastroenterology clinics. Normally, the approach in clinical practice when evaluating GI symptoms entails the screening for organic diseases, particularly when alarming signs are present, such as weight loss, bloody stool, abdominal masses, lymphadenopathy, and anemia [4]. The current literature shows that the presence of alarm features is related with a 5–10% risk of organic condition (inflammatory, neoplastic, allergic, autoimmune, etc.) [5]. However, in most cases, a thorough work-up will result in negative serologic, imaging, and endoscopic examination. In such cases, an “over-investigation” by various specialties (such as family medicine, internal medicine, surgery, etc.) should be avoided. Instead, a referral to a specialty lab in gastrointestinal motility and function could be of more benefit to evaluate the etiology of symptoms and improve care.

In recent years, substantial progress had occurred in technologies used to investigate GI motility and function [6,7]. An analysis by the International Working Group for Disorders of Gastrointestinal Motility and Function found that the routine implementation of these diagnostics would find clinically relevant pathologies that may direct management [8,9]. High resolution manometry (HRM) nowadays is considered the gold-standard method for the assessment of esophageal peristalsis and the function of the LES [10]. The advent of the HRM catheters has enabled a continuous representation of esophageal pressures and has facilitated a more accurate evaluation of esophageal body and sphincters’ function. Intraluminal impedance is an important addition to HRM catheters and enables objective assessment of bolus transit through the esophagus [6,7]. This is performed by measuring the resistance to electrical current conduction on impedance electrodes spanning the length of the HRM catheter.

Significantly, the evolution of HRM has permitted the introduction of the Chicago Classification (CC), which is the accepted working algorithm for analyzing and interpreting HRM studies. The CC divides esophageal motility disorders into major disorders (such as achalasia, esophagogastric outflow obstruction, and absent contractility) and minor motility disorders (such as ineffective peristalsis). Major motility disorders are usually pathological, cause symptoms, and require therapy, whereas minor disorders can be found in healthy persons, do not always cause symptoms, and generally do not require specific treatment. The standard protocol for HRM performance includes ten swallows of 5 mL of water. Additionally, provocative testing such as rapid water swallows and solid swallows have further improved the diagnostic yield of HRM and have recently been added to standard protocols [11].

More recently, the endoluminal functional lumen imaging probe (EndoFLIP) has been introduced and implemented in clinical practice. EndoFLIP represents a modern technology, performed under sedation, to assess function of the esophagogastric junction by using a special balloon designed to measure distensibility [12]. Additionally, EndoFLIP has been used to evaluate disorders of the pylorus.

Improvements in fluoroscopic exams, such as the video fluoroscopic swallowing exam (VFSE), timed barium swallow (TBS), and timed barium surface area measurement, have further enriched the diagnostic battery which is available when investigating esophageal symptoms such as dysphagia [13].

Gastroesophageal reflux disease (GERD) is very prevalent in the general population, may lead to severe symptoms, and negatively impacts quality of life [14]. In clinical practice, most cases of GERD are diagnosed based on clinical history and response to a trial of antisecretory therapy. Nonetheless, current evidence points towards poor association between clinical symptoms of GERD and true pathological reflux [15]. Only 30% of patients with GERD will undergo a diagnostic endoscopy to evaluate for esophagitis or Barrett’s esophagus. According to the Lyon consensus, pathological GERD could be established when acid exposure time (AET) is >6%/24 h on a pH study [16]. When the diagnosis of GERD is inconclusive, additional data may support the diagnosis including the number of refluxes in 24 h, the symptom association indexes, and novel metrics from impedance-pH monitoring such as the mean nocturnal baseline impedance (MNBI) and post-reflux swallow-induced peristaltic wave (PSPW) index [17,18].

Remarkably, artificial intelligence (AI) is being introduced in esophagology to aid in the evaluation of GERD, eosinophilic esophagitis, and motility disorders. Several AI models have been developed to autonomously extract and analyze pH-impedance tracings. This method has shown acceptable specificity and sensitivity to diagnose GERD [19].

Presently, scintigraphy of a solid egg meal using Technetium-99 m is considered the gold standard for gastroparesis diagnosis [20]. However, breath tests and wireless motility capsules may be substitutes for scintigraphy in the evaluation of gastric emptying [20].

The anorectal region is associated with many symptoms, including constipation, defecatory disorders, incontinence, and pain. High-resolution anorectal manometry (HRAM) is the standard modality used in clinical practice to objectively assess anal and rectal sensory and motor function [21]. Recently, the London classification was published. It is a consensus guideline written by an international anorectal physiology working group that proposes a practical standardized protocol for the performance and analysis of anorectal manometry [21]. The HRAM test protocol should include evaluation of rectoanal reflexes, anal tone and contractility, rectoanal coordination, and rectal sensory function.

Other complementary tests, such as the balloon expulsion test, transrectal ultrasound (TRUS), defecography, and pelvic magnetic resonance (MRI), are also used in clinical practice to assess anorectal and defecatory function [22]. Fecobionics is a 10 cm-long simulated stool device that permits active testing of several anorectal functions during defecation including pressure, surface area, and impedance. Patents can report symptoms as well during testing with fecobionics. The clinical utility of the fecobionics device in clinical practice is still evolving [23,24].

In summary, there have been many recent advances in the diagnostics and technologies used to assess GI motility and functional disorders. These advances have improved our understanding of disease pathogenesis and thus improved management. We believe that this Special Issue in the journal *Diagnostics* will aid in shedding light on new advances in diagnostic testing within the field of GI motility and function. Additionally, this Special Issue will increase the visibility of this specialty field among a wide range of medical practitioners, hopefully improving communication between other clinicians and neurogastroenterologists.

## Data Availability

Data is available from author upon request.

## Abbreviations

gastrointestinal—GI; upper esophageal sphincter—UES; lower esophageal sphincter—LES; gastroesophageal reflux disease—GERD; high-resolution manometry—HRM; Chicago Classification—CC; endoluminal functional lumen imaging probe—EndoFLIP; video fluoroscopic swallowing exam—VFSE); timed barium swallow—TBS; mean nocturnal baseline impedance—MNBI; post-reflux swallow-induced peristaltic wave—PSPW; High resolution anorectal manometry—HRAM.
